# A Novel Internet of Energy Based Optimal Multi-Agent Control Scheme for Microgrid including Renewable Energy Resources

**DOI:** 10.3390/ijerph18158146

**Published:** 2021-07-31

**Authors:** Bilal Naji Alhasnawi, Basil H. Jasim, Zain-Aldeen S. A. Rahman, Josep M. Guerrero, M. Dolores Esteban

**Affiliations:** 1 Electrical Engineering Department, University of Basrah, Basrah 61001, Iraq; hanbas632@gmail.com (B.H.J.); as.zain9391@stu.edu.iq (Z.-A.S.A.R.); 2 Department of Electrical Techniques, Qurna Technique Institute, Southern Technical University, Basra 61016, Iraq; 3 Center for Research on Microgrids (CROM), Department of Energy Technology, Aalborg University, 9220 Aalborg, Denmark; joz@energy.aau.dk; 4 Civil Engineering Department, Hydraulics, Energy and Environment/Profesor Aranguren 3, Universidad Politécnica de Madrid (UPM), 28040 Madrid, Spain

**Keywords:** renewable energy resources, diffusion algorithm, IoT, IEEE microgrid, cooperative control, cloud platform

## Abstract

The increasing integration of Renewable Energy Resources (RERs) in distribution networks forms the Networked Renewable Energy Resources (NRERs). The cooperative Peer-to-Peer (P2P) control architecture is able to fully exploit the resilience and flexibility of NRERs. This study proposes a multi-agent system to achieve P2P control of NRERs based Internet of Things (IoT). The control system is fully distributed and contains two control layers operated in the agent of each RER. For primary control, a droop control is adopted by each RER-agent for localized power sharing. For secondary control, a distributed diffusion algorithm is proposed for arbitrary power sharing among RERs. The proposed levels communication system is implemented to explain the data exchange between the distribution network system and the cloud server. The local communication level utilizes the Internet Protocol (IP)/Transmission Control Protocol (TCP), and Message Queuing Telemetry Transport (MQTT) is used as the protocol for the global communication level. The effectiveness of the proposed system is validated by numerical simulation with the modified IEEE 9 node test feeder. The controller proposed in this paper achieved savings of 20.65% for the system, 25.99% for photovoltaic, 35.52 for diesel generator, 24.59 for batteries, and 52.34% for power loss.

## 1. Introduction

Renewable energy is going to be an important source for power generation in the near future because we can use these resources again and again to produce useful energy. The energy resources are normally classified as fossil resources, renewable, and nuclear energy resources. Different renewable energy resources, such as hydropower, wind, solar, biomass, ocean energy, biofuel, geothermal, etc., provide 15–20% of the total world’s energy. The world is going to turn into a global village due to more requirement of energy due to fast-growing population, which leads to the use the fossil fuels such as coal, gas, and oil to fulfil the energy requirement, which creates unsustainable situations and many problems such as depletion of fossil fuels, environmental and geographical conflicts, greenhouse effect, global warming, and fluctuation in fuel prices. Due to environment-friendly and less emission of gases from renewable energy, it is considered as sustainable energy; also supported for the society from each dimension: economic, social, and environmental [1].

Microgrids may be a prospective power system that addresses the renewable energy technologies (RET) accompanying the necessary growing deployment of distributed energy resources (DER) [2].

The micro-grid has a hierarchical control system consisting of internal/primary, secondary, and tertiary control levels [2,3]. The micro grid is connected to the main grid during normal operation. But, in the event of a disturbance, it will change to autonomous mode. Therefore, it is crucial for a stable and economically effective micro grid operation to have an appropriate control scheme, which can keep the voltage/frequency stable and maintain active/active power-sharing. Both voltage and frequency of a micro grid are dictated by a main grid in a grid connected mode, however, in an islanded mode, primary control takes the responsibility of the voltage and frequency control [4]. Operation of a micro grid with a primary control alone, in an islanded mode, causes the steady-state load-dependent voltage and frequency deviations, giving rise to power quality problems and deteriorating the healthy operation of MG. Thus it must compensate for such variations with the use of a second additional control to revert voltage and frequency to the nominal values (i.e., standard or reference) of each distributed generator unit [5,6,7,8].

The secondary control approaches include centralised control [9], decentralised control [10], and distributed control [11]. Inspired by the cooperative Multi Agent Systems (MAS) control concept the distributed control concept. It depends on the communication between local authorities who can exchange information via a local communication network with neighbouring agents [12]. The distributed generator unit in the micro grid is regarded as the agent capable of communicating with its neighbouring agents through a sparse communication network. Thus, a micro grid behaves as a MAS.

The traditional secondary microwave control structure uses a centralized system in which each unit is connected to all the rest of the unit bi-directionally. This controller problem is gathering comprehensive information in a system that requires costly and complex network communications, lacks flexibility, and is prone to single point failure. This means that the centralized control process stops working regardless of whether or not a single unit breaks down or loses connections to the remaining network due to failure of communication links [13]. On the other hand, the main advantage of the distributed check method is that the unit is not required for communications in the communication network with all other units, thereby enhancing the reliability of the entire micro network operation and reducing bandwidth and communication network cost requirements. Because of this attribute, distributed cooperative control systems are popular for promising micro grid application solutions [1]. 

Therefore, in this paper, the researchers developed a new decentralized cooperative peer-to-peer approach for coordinating the multi Renewable Energy Resources (RERs) operation in distribution networks.

The Internet of Energy (IoE) refers to a paradigm that connects different digital, real, and virtual devices to intelligent environments via network information. It applies in many fields, including transport, energy, and urban areas. Energy Internet is considered a smart microgrid revolutionary network. In the energy and power sectors, it is regarded as an overall IoT application. The Energy Internet consists of different techniques and components, which are summarised into three groups, i.e., (i) system of power, (ii) system of communication, and (iii) control systems. In one study, the researchers stated that the Energy Internet’s cross-disciplinary nature had presented several opportunities and challenges, which have to be investigated further and validated [14].

It was noted that the MGs act as primary building blocks in an Energy Internet since they can be operated in grid-connected and islanded modes [15]. The droop based primary control can be used for autonomous power sharing among all connected distributed generations. An islanded MGs’ secondary control feature allows voltage/frequency restoration while maintaining precise power sharing among the connected distributed generations [16]. Furthermore, the tertiary control helps in the optimal operation of the microgrids (MGs) [17]. In the hierarchy, tertiary control helps determine optimal dispatch values, which are dependent on renewable and load forecasting. Regarding the dispatch intervals, both the primary and the secondary controls are operated for sharing the actual power deviation taking place from the dispatch values. A consensus algorithm-based secondary control and the distributed optimisation algorithm-dependent tertiary controls have garnered a lot of research attention owing to their increased flexibility and resilience compared to the centralised control [18,19]. Furthermore, the implementation of a distributed algorithm is dependent on Multi-Agent System (MAS), wherein multiple subsystems/agents interact with one another with the help of sparse communication networks [20].

To the best of the authors’ knowledge, achieving reactive power/active power sharing and voltage and frequency regulation, with preserved local information privacy, is still the open question. To this end, this paper presents a distributed consensus-based method to achieve reactive power, active power sharing and voltage and frequency regulation in MG. First, the original control problem is transformed into an equivalent active power reference generation problem, which can be solved by obtaining the global active power utilization level. Further, a distributed diffusion algorithm is proposed to acquire this global variable. In addition, this study objectives to offer potential solutions for following situations: (i) distributed controllers may neither be located at the same location as distributed generations nor have a proprietary communication network. A remote control of microgrids by the Internet, taking communication latency into consideration, is required. (ii) For microgrids governed via multi agent systems, each agent or sub-multi agent systems can be practically owned via different stakeholders who could cooperate or work independently. (iii) With the features in Internet of Things and renewable system, the number of controllable units in microgrid is dramatically increasing. Any distributed control system scalability to withstand increasing numbers of distributed generations is an issue worthy of exploration.

### 1.1. Related Works

Active power-sharing traditionally takes place through primary control. The centralised controller is then used to offset drop control deviations in frequency [21,22]. There is no flexibility in the centralised control structure, and there can be a single failure. The literature therefore reports distributed control algorithms [23]. The information shared by the distributed controllers over a sparse communication network can be used to achieve active power sharing and frequency control [24]. However, the distributed generations are transmitted directly to their neighbours without protection of their privacy or sensitive local data such as power outputs, usage levels, power capacity, etc.

In [25], coordinated controls were proposed, including for different distributed energy storage systems, to equalize charge status. A secure cloud-based multi-agent platform is not, however, investigated. In [26], the Combination of Communication Technology and Hierarchical Control Method proposed a coordinated method for the assessment of the state-of-the-art balance in Alternating Current (AC) MG. However, the proposed control structure will inevitably invalidate intact high level control functions. In [27], the authors proposed an efficient distributed control method for the synchronisation in the Island micro grid of several Renewable Energy Resources (RERs). The secondary control technique is developed to remove deviations in frequency and to ensure a certain time-efficient power sharing. Within a limited time frame, the proposed end time controller allows the unconnected design for the voltage control and an alternate time frame for reactive power sharing. However, the authors do not consider the graph network for data and information transfer between the MG connect agents.

In [28], the authors suggested a distributed iterative learning environment to address Direct Current (DC) microgrid’s current/voltage sharing problem. The optimal control method, which is further determined by using the iterative value algorithm, was derived in game theory. An adaptive dynamic programming architecture and algorithm were developed to share current while simultaneously changing the DC bus’s voltage to its rated value. However, active and reactive power sharing is not investigated. In [29], an MG isolated composed by parallel connected inverters from multiple voltage sources was analysed by the researchers. The primary control was integrated into every inverter by internal voltage and current systems with PR trimmings, Virtual Impedance, external voltage, and frequency drops controllers. A secondary frequency restoration function has been implemented by the investigators. This contributes to the implementation of the diffusion algorithm including a frequency control and a single communication network delay. However, a secure cloud-based platform for multi-agents is not investigated. In [30], the authors proposed a split multi-agent finite time control approach with a balance of load delay and voltage restoration in the battery’s DC MG. Theoretically, for each device, delays can be different and endless. The linearization feedback approach is used with the input time delays in dual integrated and single integration systems in order to transform charging and voltage recovery problem. However, the distributed control for multi agent system governed microgrids in Internet of Energy is not investigated.

In order to meet load demand and protection demand, the authors in [31] created a Hybrid control system, based on a multiagent system event, which utilizes online supplies of renewable energy. However, there is no study of the active and reactive power sharing. In [32], the authors suggested a new control method for voltage/frequency restore approach based on the consensus algorithm and proposed method implemented in island microgrid systems (MGs). However, a secure cloud-based platform for multi-agents is not investigated. The authors proposed a diffused method for coordination control of hybrid microgrids in [33]. The method proposed regulates accurate dc current and reactive power shares between distributed microgrid generators, maintains power-sharing between the two microgrids and restores a DC voltage, and the AC frequency, to their rated values. However, the authors do not consider the graph network for data and information transfer between the MG connect agents. In [34], researchers suggested a collapsing distributed and hierarchical cooperative control method for microgrid cluster, including distributed layer generation, micro grid layer, and cluster layer controls for MG. The distributed generation layer control regulates each distributed unit’s current/tension locally. The control of the microgrid layers for each microgrid is performed to positively manage distributed generating units via several small communication networks. The control of the Cluster-Layer co-ordinates micro grids on the basis of a more advanced peer-to-peer communication interface between micro-grid-agents. However, the distributed control for multi agent system governed microgrids in Internet of Energy not investigated. In [35], a new distributed multi-agent framework based on the cloud layer computing architecture is developed for real-time microgrid economic dispatch and monitoring. In [36], the Time of Use (ToU) model is proposed to define the rates for shoulder-peak and on-peak hours. A two-level communication system connects the microgrid system, implemented in MATLAB, to the cloud server. In [37], the researchers proposed a multi-agent and multi-layer architecture for acquiring the P2 P control of the MGs. Here, the control framework was distributed entirely and it contained three control layers that were operated in every MG. For the primary control, the researchers adopted a droop control for every MG-agent to carry out a localized power-sharing. The researchers proposed a distributed diffusion for each secondary control that helped in voltage/frequency restoration and arbitrary power-sharing amongst the microgrid. However, a cloud-based platform for multi-agents is not investigated.

The existing technical studies do not address the following limitations.

- The critical bus voltage, subject to distributed secondary voltage regulation, must be restored to ensure continuous operation of sensitive loads. Literature [38] provides critical bus voltage restoration, but it doesn’t simultaneously maintain accurate reactive-power sharing among units of Renewable Energy Resources (RERs) [39].
- To the best of our knowledge, a behaviour and analysis of distributed secondary control, when the AC side voltage of a distributed generator unit reaches to its limit, has not been reported.
- Literature [40,41,42] assume purely inductive networks for small signal dynamic analyses of the distributed secondary frequency controller [42] and the distributed secondary voltage controller [40,41]. For a practical micro grid system, especially the low voltage (LV) micro grid system
- The active power, reactive power-sharing simultaneous regulation are not investigated. Second, the distributed diffusion control method for multi agent governed microgrids in the Internet of Energy has not been studied. This motivates us to provide a novel approach that enables the group play and plug feature, such that microgrids with multiple multi agent, owned by different stakeholders, can be flexibly controlled.

### 1.2. Paper Contribution

The present developments and limitations of the literature have led the researcher to propose a fully distributed diffusion based control system for the achievement of several objectives. The chief contributions of this paper are summarised as:

1. The peer-to-peer control architecture considering multi-agent and multi-layers interaction is introduced for a distribution networks in the Energy Internet, which has not been reported in the past.
2. This paper summarises the findings of researchers in distributed control design of RERs devices in microgrid to provide ancillary services, including equal reactive power sharing, equal active power-sharing between RES units, and controlling the load in both islanded mode and grid-connected mode.
3. An IoT-based communication protocol including specifications such as MQTT is proposed. This improves system flexibility. The proposed system offered analytics and business intelligence (BI), which allowed the researchers to gain insights on data collected by visualizing dashboards and reports. Additionally, the use of large data-based data storage technologies enabled the system’s scalability at the national level. This provided energy-efficiency strategies for the home owners and the utility companies.

## 2. Proposed System Description

**Figure 1:**
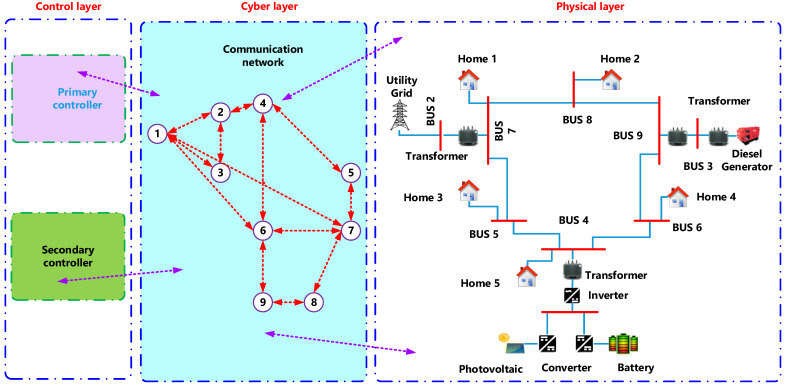
IEEE 9 bus system, with renewable energy sources and communication system.

Here, the researchers considered that the RESs consisted of the communication and control agents on the Internet of Energy realm, as described in Figure 1. The physical components of a general microgrid included the inverter-interfaced Renewable Energy Resources [Such as photovoltaic, wind turbine, and energy storage systems], dynamic and static loads, and the diesel generators. It was noted that a framework controlled the RESs in a microgrid, wherein one MAS agent managed every RESs. The MAS agents communicate by Local Area Network (LAN) and can access the Internet for remotely controlling the microgrid via the cloud servers. In the Energy Internet, every distributed generator/microgrid was managed by various stakeholders and their controllers on the MAS/agents differed from MG components. It was expected that number of the distributed generator and MG agents could be changed online. Hence, a remote, flexible, and distributed control and implementation framework were necessary. Figure 1 presents a proposed system.

A smart grid would need an effective measuring and communication system to continuously track the power and cost profile and regularly quantify power losses. There are several stages of data processing.

This work contains measurement units (MU) for every distribution network bus. MU is MATLAB modelling. Power and cost information is sent to the control centre regularly at a fixed time. The control centre is designed as a virtual data management and analysis platform. One approach to communication, relating to the device topology proposed, is considered. The case takes a Cloud approach, which sends its measured data directly to the cloud by any MU connected to the corresponding feeder bus, as illustrated in Figure 1.

The data transfer among the MATLAB software package and the open-source IoT framework ThingSpeak are used to model proposed communication architectures. ThingSpeak was chosen for the simulation of real-time cloud communication due to its following benefits [43]:

1. ThingSpeak Cloud IoT platform data aggregation, tracking and analysis. In the smart grid model, the power profile is monitored on multiple ThingSpeak channels in real-time and depicted graphically.
2. Security: The Username and password allow user authentication while each channel is equipped with its ID and accessible (see by other users). There are two keys in each channel for the application programming interface. A randomly generated read key and write key of the API. These keys can save or retrieve information from each channel over the Internet or LAN.
3. It facilitates the double-way flow of data between the user and virtual device and allows data and remote control to be exchanged in real-time. The MATLAB Desktop Toolbox offers communication between the simulated feeding model and the ThingSpeak IoT platform.
4. Communication network enabling for the data transmission over the Internet between MATLAB and ThingSpeak.
5. Allows importing, exporting, analysing, and viewing data on multiple platforms and their fields simultaneously,

## 3. Renewable Energy Resources (RERs)

### 3.1. Photovoltaic Cell Modeling

**Figure 2:**
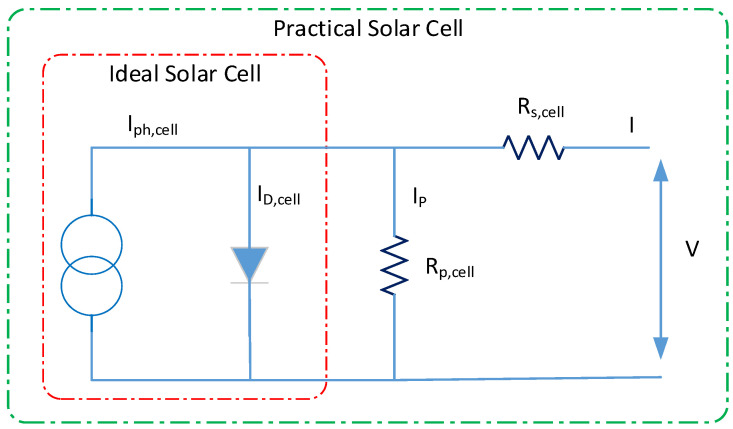
Equivalent circuit of photovoltaic.

**Figure 3:**
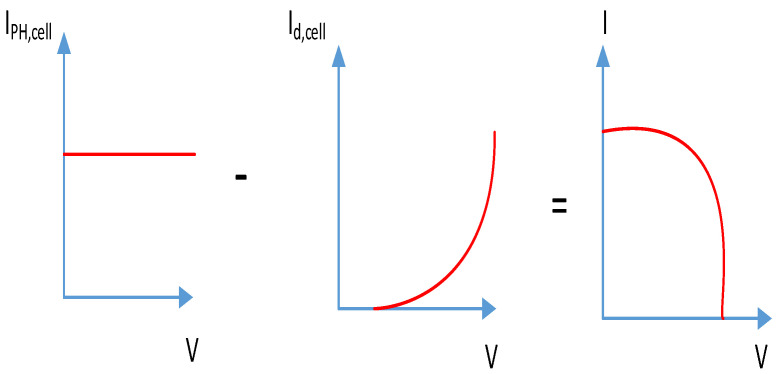
Voltage and current of photovoltaic.

Figure 2 shows a single photovoltaic cell diode system based upon which current source, diode, resistance series, and parallel resistance are represented. In the Figure 3 Illustration, the photovoltaic cell current-voltage characteristics are described in the mathematical equation standard [44]:

(1) $$I=I_{\mathrm{ph};\mathrm{cell}}-I_{0;\mathrm{cell}}\;\left[exp\left(\frac{q(V+{\mathrm{IR}}_{s;\mathrm{cell}}\;}{\mathrm{akT}}\right)-1\right]-\frac{V+{\mathrm{IR}}_{s;\mathrm{cell}}}{R_{p;\mathrm{cell}}},$$

where: $I_{\mathrm{ph};\mathrm{cell}}$, is a current (A) of photovoltaic; $I_{0;\mathrm{cell}}$, is a saturation current of a photovoltaic; *T* is a temperature of a diode; *k* is a constant of Boltzmann ($1.38\times 10^{-23}\;J/K$); $R_{p}$, is a parallel resistance of PV (Ω); $R_{s}$, is a series resistance of PV (Ω), V is a thermal voltage.

### 3.2. Photovoltaic Modeling

**Figure 4:**
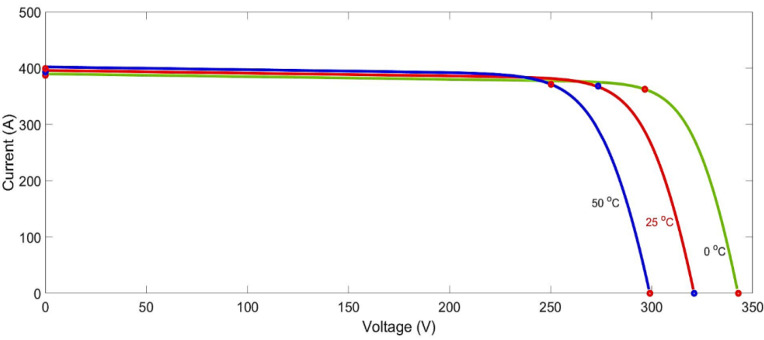
Current-voltage, photovoltaic module curves at different temperatures and permanent insolation levels.

The photovoltaic module consisting of PV cells joined in a parallel and series shapes is mentioned above. Therefore, Equation (1) derives from the mathematical standard and the PV module description of its I-V characteristic [44]:

(2) $$I=I_{\mathrm{PH}}-I_{O}\left[\exp\left(\frac{V+{\mathrm{IR}}_{S}}{a\;\mathrm{Vt}}\right)-1\right]-\frac{V+{\mathrm{IR}}_{S}}{\mathrm{Rp}},$$

where: Vt is a thermal voltage, $I_{\mathrm{PH}}$ is a photocurrent (A), $R_{S}$ is a series resistance, $I_{O}$ is a reverse leakage current, $R_{p}$ is a parallel resistance. The Equation (2) produces voltage and current curve as indicated in Figure 4.

The PV module’s photocurrent ($I_{\mathrm{PH}}$) is determined by the amount of solar radiation falling on modulus and photovoltaic cell temperature that fits the equation below [44]:

(3) $$\mathrm{Iph}=\frac{G}{\mathrm{Gn}}\left(I_{\mathrm{ph};n}+\mathrm{Ki}\Delta T\right)$$

where: $I_{\mathrm{PH}.n}$ is a photocurrent; $G_{n}$ is a irradiance

(4) $$V_{\mathrm{oc}}=V_{\mathrm{oc};n}+K_{v}\Delta T$$

where: $K_{v}$ is a temperature coefficient, $V_{\mathrm{oc};n}$ is open circuit voltage.

(5) $$I_{o}=\frac{I_{\mathrm{sc};n}+K_{i}\Delta T}{\exp\left(\frac{V_{\mathrm{oc};n}+K_{v}\Delta T}{{a\;V}_{t}}\right)-1},$$

where $I_{\mathrm{sc};n}$ is a short-circuit current.

#### System of Energy Storage

The system of battery storage stores excess energy generated by generation of renewables. In the event of energy shortages from the renewable energy systems batteries will be discharged so as to meet demand for load. Simple dynamics of batteries are modelled, such as [44,45]:

(6) $$SOC_{\mathrm{bat}}=100\left[1-\left(\frac{1}{Q_{\mathrm{bat}}}\cdot \int_{0}^{t}i_{\mathrm{bat}}\left(t\right)dt\right)\right],$$

(7) $$B_{AH}=\frac{1}{3600}\int_{0}^{t}i_{\mathrm{bat}}\left(t\right)dt,$$

where $Q_{\mathrm{bat}}$ is a maximum batteries capacity (A h), $SOC_{\mathrm{bat}}$ is a batteries state of charge (%), $B_{AH}$ is a battery ampere-hour and $i_{\mathrm{bat}}$ is the battery current. 

### 3.3. Diesel Generator of Disterbuted Network

In the micro grid, this diesel generator balances power and charge. A diesel engine, a control system, an exciting system, and a simulated machine are included in the models. Diesel engine and the model system governor are combined with speed inputs into one unit (in p.u.). The mechanical capacity of the diesel motor is the block output. The control function is modelled as follows [46]:

(8) $$H_{c}=\frac{k\left(1+T_{3}S\right)}{1+T_{1}S+T_{1}T_{2}S},$$

where $T_{1}$, $T_{2}$ and $T_{3}$ are regulator time constants, *k* is a proportional gain and. The actuator transfer function is as:

(9) $$H_{a}=\frac{1+T_{4}S}{\left[s\left(1+T_{5}S\right)\left(1+T_{6}S\right)\right]},$$

where $T_{4}$, $T_{5}$ and $T_{6}$ are actuator time constants. An excitement system is represented by the following transfer function for the synchronous machine.

(10) $$\frac{V_{fd}}{V_{ro}}=\frac{1}{K_{s}+ST_{e}},$$

where $V_{ro}$ is a regulator’s output, $V_{fd}$ is a exciter voltage, $T_{e}$ is time constant (seconds), $K_{s}$ is the gain. 

### 3.4. Problem Formulation

This paper considered an MG with N controllable distributed generator (indexed as I = 1, 2, …, N.). The MGs electrical network is presented using an elaborate weighted graph, $T=\left(V_{T},\;E_{T}\right)$, wherein the nodes $V_{T}=\left\{v_{1},\;v_{2},\ldots v_{N}\right\}$ represented the buses (RES) and edges, $E_{T}\subseteq V_{T}\times V_{T}$, represented line connections [47].

### 3.5. Primary Control of Inverter

**Figure 5:**
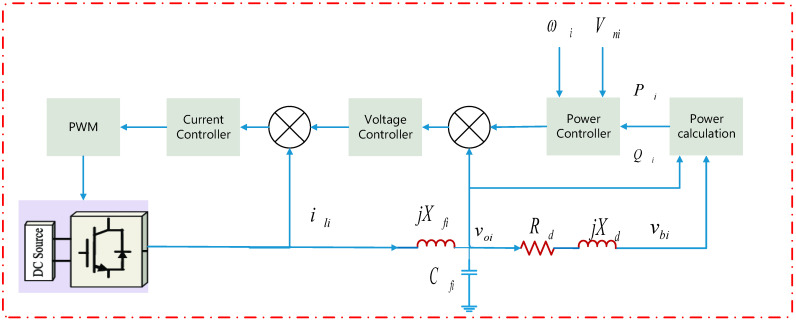
Schematic of control.

The basic graph for Renewable Energy Resources (RERs) connected via AC/DC converters and LCL filters is shown in Figure 5. The proposed primary control is shown in Figure 5 [48].

(11) $$\omega_{ni}=\omega_{i}+m_{Pi}P_{i}$$

(12) $$V_{ni}=V_{oi}+m_{Qi}Q_{i}$$

where $\omega_{ni}$ and $V_{ni}$ are the nominal set points for frequency and voltage, $\omega_{i}$ and $V_{oi}$ are the frequency and voltage of DG i, $m_{Pi}$ and $m_{Qi}$ are the frequency and voltage magnitude droop coefficients of DG i, respectively.

### 3.6. MASs Communication

**Figure 6:**
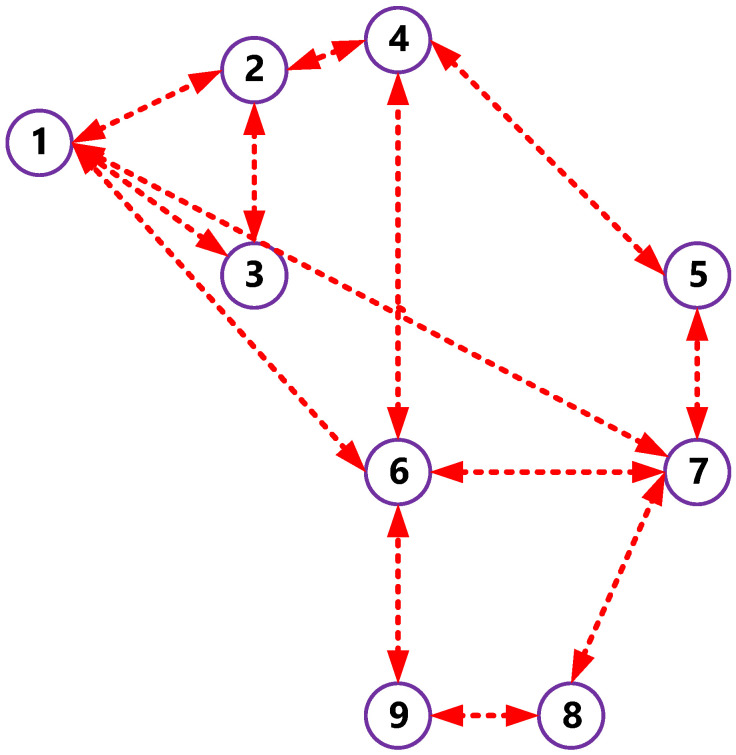
The information exchange graph among the connected agents.

The communication networks of microgrid having N agents was represented using a graph: $G=\left(V_{G},{ℰ}_{G}\right)$ having a defined set of nodes $V_{G}=\left\{v_{1},v_{2},\;\ldots ,\;v_{N}\right\}$ and edges ${ℰ}_{G}\subseteq V_{G}\times V_{G}$. All nodes presented in the graph G(agents) showed a one-to-one correspondence to the units in the graph T (renewable energy resources). Furthermore, edges in G, which represented the communication links for the data exchange, differed from electrical connection seen in T. In addition, set of neighbors described in the ith node of G was represented by $N_{i}=\left\{v_{j}\in V_{G}:\left(v_{i},v_{j}\right)\in {ℰ}_{G}\right\}$. The researchers represented the adjacency matrix as $\left[a_{ij}\right]\subseteq R^{n\times n}$ [11]. Here, the term $a_{ij}$ represented the information that was exchanged between the units i and j, wherein $a_{ij}=1$ when units i and j were connected with the edge ($v_{i},v_{j})\in {ℰ}_{G}$, else $a_{ij}=0$. The researchers represented the Laplacian matrix as $L=\left[l_{ij}\right]\subseteq R^{n\times n}$ where each element $l_{ij}=\sum_{i=1}^{n}a_{ij}-a_{ji}$. They described the pinning matrix as $G=diag\left[g_{i}\right]\subseteq R^{n\times n}$ and $g_{i}=1$ when the RER/agent could access the references $\omega^{ref}$ and $V^{ref}$, else $g_{i}=0$. Figure 6 presents the data exchange between the controllers.

### 3.7. Proposed Secondary Distributed Controller 

The chief objective of this section is to add to droop controller of renewable energy resources a secondary controller. In order to control the frequency and voltage of the system in a common connecting point, the controller receives information from neighbouring RES and sharing power between appliances. Moreover, a virtual leader can be assigned to one RES or a couple of storage devices on the system. The leader has the tension and frequency setpoints of the system and shares the information with his neighbouring storage units. The RES model needs to be developed in order to develop such a control design. A simplified RES model has been developed in our recent work. The model reflects the dynamics of the DC and the RES active power. The RES dynamics of devices in smart grids can be precisely incorporated into this model. The RER device dynamics can be displayed [49]:

(13) $$\omega_{i}=\omega_{i}^{nom\;}-D_{i}^{P}P_{i}$$

(14) $$\left|V_{i}\right|=\left|V_{i}^{nom}\right|-D_{i}^{Q}Q_{i}$$

(15) $${\dot{E}}_{i}=\frac{-D_{i}^{P}}{3600}P_{i}$$

(16) $${\dot{P}}_{i}=u_{i}^{P}$$

For the development of such a simplified model, voltage controller and current controller dynamics are supposed to be much faster than a droop controller, so its dynamics may be ignored. $u_{i}^{P}$ for distributed active power-sharing is input in the above-mentioned model, and $D_{i}^{P}$ reflects RESs heterogeneity. To ensure the equality of power sharing, $D_{i}^{P}$ $P_{i}$ should be controlled by batteries in order to increase power sharing in a RES with higher capacity (lower drop-in gain, $D_{i}^{P}$). This study regulates nominal voltage and the frequency of adjacent RES units in order to minimize communications between RERs. The control design will require only nominal frequencies $\omega_{i}^{nom}$ and the nominal tension $\left|V_{i}^{nom}\right|$ of its adjacent devices, and thus, only the frequency and voltage signals from neighbouring RESs. These inputs include $\left|{\dot{V}}_{i}^{nom}\right|=u_{i}^{V}$, ${\dot{Q}}_{i}\&=u_{i}^{Q}$ and ${\dot{\omega }}_{i}^{nom}=u_{i}^{\omega }$. The overall dynamics of the ith is formulated as [49]:

(17) $${\dot{E}}_{i}=\frac{-D_{i}^{P}}{3600}P_{i}$$

(18) $${\dot{P}}_{i}=u_{i}^{P}$$

(19) $$\left|{\dot{V}}_{i}^{nom}\right|=u_{i}^{V}$$

(20) $${\dot{Q}}_{i}=u_{i}^{Q}$$

(21) $${\dot{\omega }}_{i}^{nom}=u_{i}^{\omega }$$

Let $\omega^{ref}$ and $V^{ref}$ be a reference voltage and frequency of RES. These references are used as external commands, which force RES to precisely converge voltage and frequency to their desired values. The respect, design of diffusion is proposed as:

(22) $$u_{i}^{P}=\frac{-C_{2}}{D_{i}^{P}}\underset{j\in N_{i}}{\sum }\left(D_{i}^{P}P_{i}-D_{j}^{P}P_{j}\right)$$

(23) $$u_{i}^{V}=-C_{3}\underset{j\in N_{i}}{\sum }\left(\left|V_{i}^{nom}\right|-\left|V_{j}^{nom}\right|\right)-C_{0}^{V}a_{0i}\left(\left|V_{i}^{nom}\right|-D_{i}^{Q}Q_{i}-\left|V^{ref}\right|\right)$$

(24) $$u_{i}^{Q}=\frac{-C_{3}}{D_{i}^{Q}}\underset{j\in N_{i}}{\sum }\left(D_{i}^{Q}Q_{i}-D_{j}^{Q}Q_{j}\right)$$

(25) $$u_{i}^{\omega }=-C_{2}\underset{j\in N_{i}}{\sum }\left(\omega_{i}^{nom}-\omega_{j}^{nom}\right)-C_{0}^{\omega }a_{0i}\left(\omega_{i}^{nom}-D_{i}^{P}P_{i}-\omega^{ref}\right)$$

Let ${\dot{\tilde{P}}}_{i}≜D_{i}^{P}P_{i}$ and ${\dot{\tilde{Q}}}_{i}≜D_{i}^{Q}Q_{i}$, then a closed-loop model of renewable energy resources with a diffusion design Equations (22)–(25) are [49]:

(26) $${\dot{E}}_{i}=\frac{-1}{3600}{\tilde{P}}_{i}$$

(27) $${\dot{\tilde{P}}}_{i}=-C_{2}\underset{j\in N_{i}}{\sum }\left({\tilde{P}}_{i}-{\tilde{P}}_{j}\right)$$

(28) $$\left|{\dot{V}}_{i}^{nom\;}\right|=-C_{3}\underset{j\in N_{i}}{\sum }\left(\left|V_{i}^{nom\;}\right|-\left|V_{j}^{nom\;}\right|\right)-C_{0}^{V}a_{0i}\left(\left|V_{i}^{nom\;}\right|-{\tilde{Q}}_{i}-\left|V^{\mathrm{ref}\;}\right|\right)$$

(29) $${\dot{\tilde{Q}}}_{i}=-C_{3}\underset{j\in N_{i}}{\sum }\left({\tilde{Q}}_{i}-{\tilde{Q}}_{j}\right)$$

(30) $${\dot{\omega }}_{i}^{nom\;}=-C_{2}\underset{j\in N_{i}}{\sum }\left(\omega_{i}^{nom\;}-\omega_{j}^{nom\;}\right)-C_{0}^{\omega }a_{0i}\left(\omega_{i}^{nom\;}-{\tilde{P}}_{i}-\omega^{\mathrm{ref}\;}\right)$$

(31) $$\tilde{P}\left(t\right)=e^{-C_{2}ℒt}\tilde{P}\left(0\right)$$

where ℒ is symmetric matrix, $\tilde{P}\left(0\right)$ is vector of initial proportional active power. $e^{-C_{2}ℒt}$ is a symmetric matrix.

(32) $$e^{-C_{2}ℒt}=U\underset{t\to \infty }{\lim}diag\left\{1,e^{-C_{2}\lambda_{2}t},\ldots ,e^{-C_{2}\lambda_{N}t}\right\}U^{T}=Udiag\left\{1,0,\ldots ,0\right\}U^{T}$$

where $\lambda_{2},\ldots ,\lambda_{N}$ are positive eigenvalues of ℒ once G is connected. On the other hand, $\mathrm{Udiag}\left\{1,0,\ldots ,0\right\}U^{T}=\left[1_{N}/\sqrt{N},0_{N},\ldots ,0_{N}\right]U^{T}=1_{N}1_{N}^{T}/N$ where the eigenvector associated with zero eigenvalue of ℒ is $1_{N}$.

(33) $$e^{-C_{2}ℒt}\tilde{P}\left(0\right)=\frac{1_{N}^{T}\tilde{P}\left(0\right)}{N}1_{N}$$

(34) $${\hat{\omega }}_{i}≜\omega_{i}-\omega^{\mathrm{ref}\;},i=1,\ldots ,N;\hat{\omega }≜{\left[{\hat{\omega }}_{1},\ldots ,{\hat{\omega }}_{N}\right]}^{T}$$

(35) $$\begin{array}{cc} {\dot{\hat{\omega }}}_{i} & ={\dot{\omega }}_{i}={\dot{\omega }}_{i}^{nom}-{\dot{\tilde{P}}}_{i}\\ & =-C_{2}\underset{j\in N_{i}}{\sum }\left(\omega_{i}^{nom}-\omega_{j}^{nom}\right)-C_{2}\underset{j\in N_{i}}{\sum }\left({\tilde{P}}_{i}-{\tilde{P}}_{j}\right)-C_{0}^{\omega }a_{0i}{\hat{\omega }}_{i}\\ & =-C_{2}\underset{j\in N_{i}}{\sum }\left({\hat{\omega }}_{i}-{\hat{\omega }}_{j}\right)-C_{0}^{\omega }a_{0i}{\hat{\omega }}_{i} \end{array}$$

Denote $D_{0}≜\mathrm{diag}\;{\left\{a_{0i}\right\}}_{i=1,\ldots ,N}$. Consequently, we obtain from (35) that

(36) $${\dot{\hat{\omega }}}^{nom\;}=-\left[C_{2}ℒ+C_{0}^{\omega }D_{0}\right]\hat{\omega }$$

**Figure 7:**
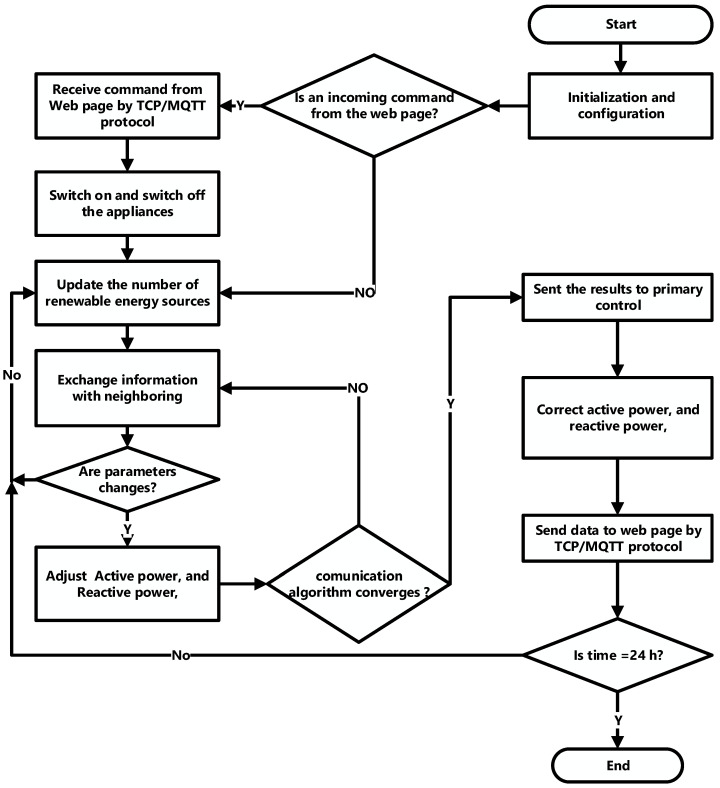
The proposed method flowchart.

If at least one of the folder is connected with a leader, e.g., $D_{0}$ is not a zero matrix, the communications graph G between the followers was shown in [49] that all the values on $C_{2}ℒ+C_{0}^{\omega }D_{0}$ of the matrix were positive in real parts for all $C_{2}>0$ and $C_{0}^{\omega }>0$. $\underset{t\to \infty }{\lim}\hat{\omega }\left(t\right)=0$. This corresponds to the battery frequency diffusion of the reference frequency $\omega^{ref\;}$. Figure 7 shows the proposed method flowchart.

## 4. Proposed Internet of Energy Communication Platform

**Figure 8:**
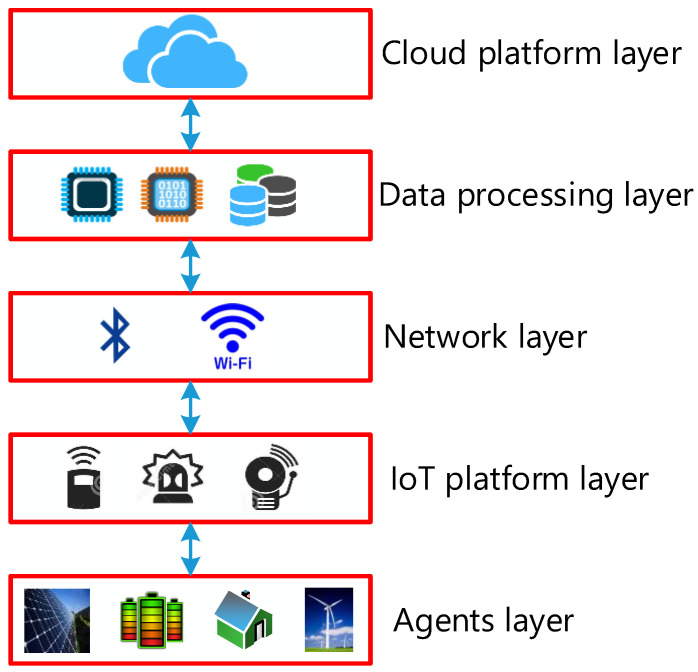
Proposed Internet of Energy platform architecture.

The decentralised controller of a smart MG helps in managing the system operating conditions if there is some disturbance. Furthermore, the IoT technology can be used for communicating between the appliances present in smart homes, central controller, or power management centres. The researchers proposed the IoT platform for collecting the data, monitoring, managing, and controlling a smart microgrid. All appliances and energy resources were integrated and connected in this platform. The major IoT platform layers included energy supply layer, network layer, energy management layer, energy appliance layer, control system layer, and the Internet of Things service layer, as presented in Figure 8.

It is a demanding job to develop an energy management distributed Energy Internet (IoE) base. The role of the platform is to (1) incorporate the micro-grid tools into the communications system and (2) link to the IoE cloud in order to track and manage the devices. The IoE communications network proposed is composed of four different layers, as defined in Figure 8. Following is a summary of each layer.

(a) Agent Layer

The device or perception layer was referred to as the layer of different components [50]. Various IoT users are included in the device layer, which comprises of smart electric vehicles, smart homes, and transportation systems, along with RERs such as FCs, MTs, and the WTs. Additionally, this layer supported different kinds of sensors for measuring the real-time environmental and physical state of the components and the actuators needed for adjusting them. Hence, WSNs and WSANs were seen to be an inseparable component of this layer.

(b) IoT platform layer

The IoT platform layer is the sensors layer. Moreover, this layer supports different types of sensors to monitor the environmental or physical condition of connected agents and to adjust them in real-time. Wireless sensor and actor network (WSANs) and wireless sensor network (WSNs) are the two pieces of the sheet that are inseparable. WSNs can be described as a number of sensors that are used to sense the environmental conditions and transmit them through a wireless network to other devices or upper layers.

(c) Network layer

Network layers can assemble the data from cloud and perception layers and then transfer it to upper layers for extra processing and storage. It can transmit the data to other smart devices for distributed functions present on component edges. A few communication topologies that are used in changed areas include WIFI, Bluetooth, Z-Wave, Zigbee, 3G/4G, LoRa, UMB, and cellular networks. These devices provide a wireless communication facility and can be used in various applications.

(d) Layer of data processing

Layer of data processing is defined as the layer which allows processing and storing a huge volume of data, which was assembled from lower layers with the help of powerful processors [50].

(e) Layer of cloud

The cloud layer stores a historical data from distributed energy resources (DERs) for the purpose of global tracking. One of the features required for IoT applications and services is to store historical data [51]. The IoE cloud layer includes virtualized servers. In addition, an application interface has been introduced with preserved historical data for each DER. A vast volume of data can be saved and maintained in the historical archive, which is supported by the application interface to the cloud infrastructure [52].

### 4.1. MQTT Knowledge

Message Queuing Telemetry Transport (MQTT) is a lightweight protocol that makes effective use of the network bandwidth with a fixed header of two bytes. The MQTT is operational on TCP and ensures that all messages are sent from agent to server.

**Figure 9:**
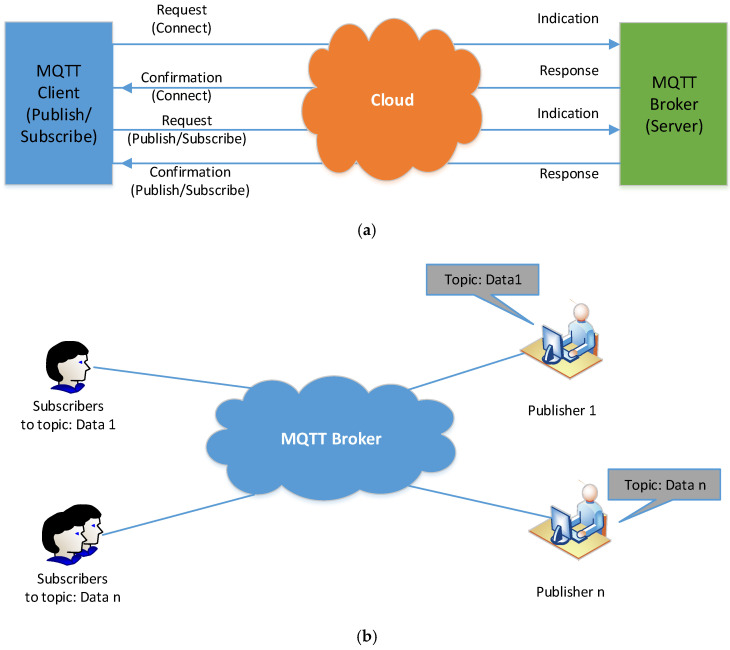
(**a**) MQTT Procedure, (**b**) MQTT Topic and Component.

Three main players, MQTT broker, MQTT publisher, and a MQTT subscriber, are included in a protocol [53]. The MQTT subscriber and publisher are indirectly linked and do not use one IP address simultaneously. MQTT Broker refers to the network gate way that filters, obtains, and distributes the publishers’ messages to the thousands of simultaneously-connected MQTT subscribers. An MQTT broker takes care of the customer authorization and initialization process necessary for communication. To publish the information, the MQTT publishers utilize custom themes for catering to their clients. The MQTT protocol did not use Metadata marking. After that, the MQTT topic management presents the metadata for a message load, which is considerable, and it can attach meaningful attributes to topic. MQTT is seen to be a string having a multi-attribute and multi-level layer. All subjects could be updated for deriving the routing data. Figure 9a presents the connection’s initialization after exchanging the control packets between the clients and brokers. It was noted that the check packets for the CONNAC, Link, PUBACK, PUBLISH, SUBSCRIBE, SUBACK, etc., comprise specific instructions regarding the theme, transmission, and the payload Quality of Service (QoS). Figure 9b presents all components of the MQTT contact.

### 4.2. Architecture of Proposed System

**Figure 10:**
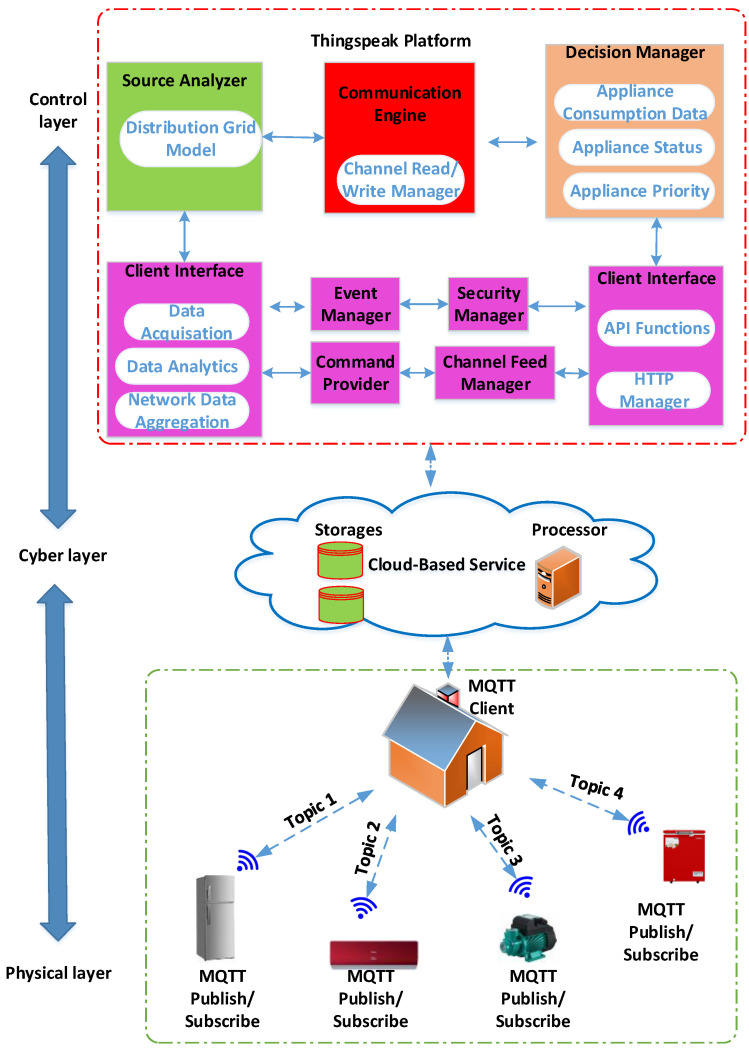
Proposed communication architecture of microgrid.

Figure 10 presents an overview of smart homes’ hierarchical platform with a cyber layer, physical layer, and control layer. Two communication layers were included in the hybrid platform. It was seen that in Layer one (local layer), the appliances in the smart building transmitted the MQTT messages to a Building MQTT Client (BMC), reported the events/measurement, and subscribed to the MQTT messages that BMC published for protection/control purpose. Layer two (global layer) represented the interaction between the cloud and BMC with the HTTP GET/POST requests’ help. In this architecture, every appliance was equipped with a Wi-Fi module connected to the local gate way. Thus, it could periodically publish the values of a dedicated and pre-defined topic. After that, BMC subscribes to different issues and posts received values to the cloud channel. Cloud data can be accessed by the cloud MATLAB interface, which implements the designed appliance resource allocation algorithm. The researchers found that if communication failure occurs in any layer, the architecture proposed is resilient (either local or global). BMC was therefore developed to operate as a local controller for all devices in the building during any communication link failure or high network latency noted. The results section highlighted this function of the BMC [11].

## 5. Result Analysis and Discussion Proposed Method

The proposed controller is tested with the micro grid, as shown in Figure 1. Here, researchers have described the simulated implementation of the distributed secondary controller, on a multi-agent system platform, in addition to their correlation with the cloud server and LAN. The multi-agent system was implemented in the MATLAB cluster connected to LAN via the network switch and connected to the cloud server via the Internet. Local communication was carried out by the TCP/IP protocol, whereas the TCP protocol conducted the communication between the cloud server and MAS. Communication between the agents was in the form of a client/server format with the help of ThingSpeak and could be configured for any network topology. In the ThingSpeak-based communication system, every agent acted as the server, which waits for the incoming messages. It can dispatch the messages to a corresponding technique since it was the neighbouring server’s client.

## 6. Access to Internet Web Page

**Figure 11:**
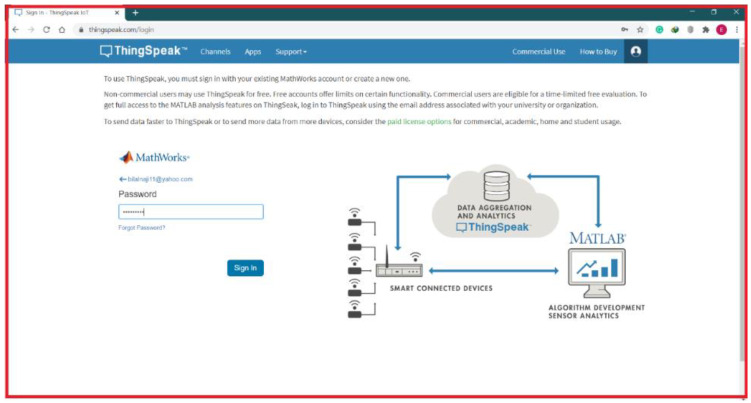
Thing-Speak platform.

In this study, the researchers carried out a simulation test, where they described and discussed the results of a decentralised power-management and control approach for micro grid in Energy Internet paradigm, which was implemented using the proposed algorithm over the cloud platform for regulating the appliances in a smart home. As noted in software communication and architecture interface, a MATLAB program was present for the Main Command and Control Unit (MCCU), which helped organize all ThingSpeak platforms. The MQTT (Mosquitto) functions as a broker and bridges the home appliance subscription and MCCU publishers’ gap. For regulating the home appliances through the MQTT gateway, the researchers used a custom code derived from the proposed MATLAB-based algorithm for its deployment. Here, the researchers designed a ThingSpeak dashboard interface, using a simple and effective user interface (UI), which allowed the homeowners to access and interact with the home energy management service over the cloud system. Figure 11 presents an internet web page that can be accessed in any internet browser after entering and providing their username and password.

This section discusses the effect of the microgrid communication system. The microgrid will exchange information in the presence of the communications device, such as load consumption and power generation. To ensure power-sharing of microgrid operating costs, the microgrid gets required power from a neighbouring microgrid. That means a communication system provides the data needed to transfer power between the microgrids, utilizing the IEEE 9 bus system in reference [54].

**Figure 12:**
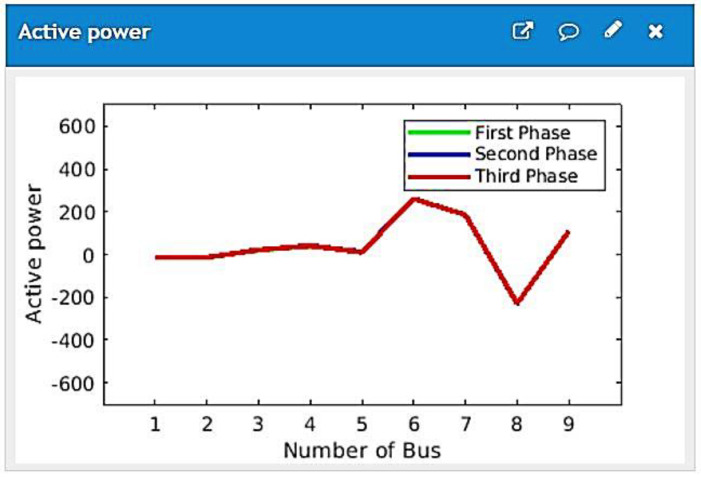
The graphical user interface of active power profile.

**Figure 13:**
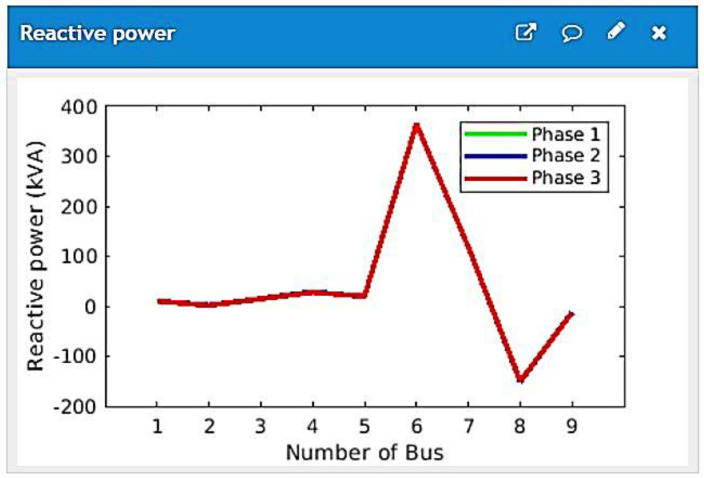
The graphical user interface of the reactive-power profile.

The experimental results noted in MATLAB for the power, voltage, and actual power of every RERs have been presented in Figure 12 and Figure 13. All renewable energy sources in the microgrid autonomously alters their power output for fulfilling load demands. Results for Scenarios indicated that a cloud server’s distributed MAS control for the remote microgrid was an effective technique. This study extracts and simulates SPR-305E-WHT-D solar. Table 1 lists these factors. Table 2. BUS generation and load parameters of IEEE 9 test system [55].

$P_{R_{i}}$ and $D_{R_{i}}$ are the nominal active power generation and the reciprocal of frequency droop gain of the inverter-based generator at bus i, respectively. $P_{L_{i}}$ and $D_{L_{i}}$ are the nominal load and the frequency coefficient of the load at bus I, respectively. $P_{R_{i}}$ = 0 and $D_{R_{i}}$ = 0 ($P_{L_{i}}$ = 0 and $D_{L_{i}}$ = 0) if no inverter-based generator (load) is connected to bus i.

Figure 12 illustrates the graphical user interface of power profile at demand scenario. Figure 13 illustrates the graphical user interface of reactive power profile at demand scenario. The proposed system’s effectiveness of proposed scheme for remote microgrid via cloud server is validated. 

## 7. Results Discussion

The results are illustrated in Figure 12 and Figure 13 Measurement of power sharing for all renewable energy resources. It is noted from the results that:

1. In both scenarios, as Figure 12 illustrates, the aims of active power sharing can be accurately achieved.
2. From Figure 13, the suggested system can realise accurate sharing of reactive power. As the control objective of power sharing is to make sure both reactive and active power sharing among generators follows renewable energy resources,

In the system’s case, the efficiency and effectiveness before applying the proposed algorithm is 0.639. After implementing the suggested MAS method, efficiency of the system is found to be 0.771. By comparing proposed algorithms with traditional method, the proposed algorithm in our work saved 20.65%.

In the photovoltaic case, the efficiency and effectiveness before applying the proposed algorithm is 0.677. Whereas after implementing the suggested MAS method, efficiency of system is found to be 0.853. By comparing proposed algorithms with traditional methods, the proposed algorithm in our work saved 25.99%.

In the diesel generator case, the efficiency and effectiveness before applying the proposed algorithm is 0.653. Whereas after implementing the suggested MAS method, efficiency of the system is found to be 0.883. By comparing proposed algorithms with traditional methods, the proposed algorithm in our work saved 35.52%.

In the battery case, the efficiency and effectiveness before applying the proposed algorithm is 0.687. Whereas after implementing the suggested MAS method, efficiency of system is found to be 0.856. By comparing proposed algorithms with traditional methods, the proposed algorithm in our work saved 24.59%.

In the power loss case, the efficiency and effectiveness before applying the proposed algorithm is 2.541. Whereas after implementing the suggested MAS method, efficiency of system is found to be 1.211. By comparing proposed algorithms with traditional methods, the proposed algorithm in our work saved 52.34%.

**Figure 14:**
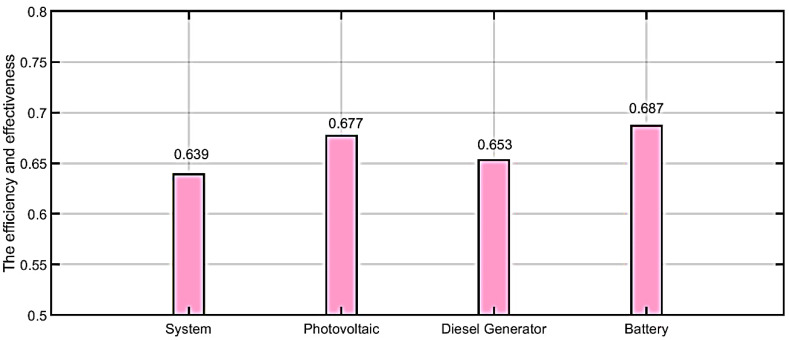
The efficiency and effectiveness without proposed method.

**Figure 15:**
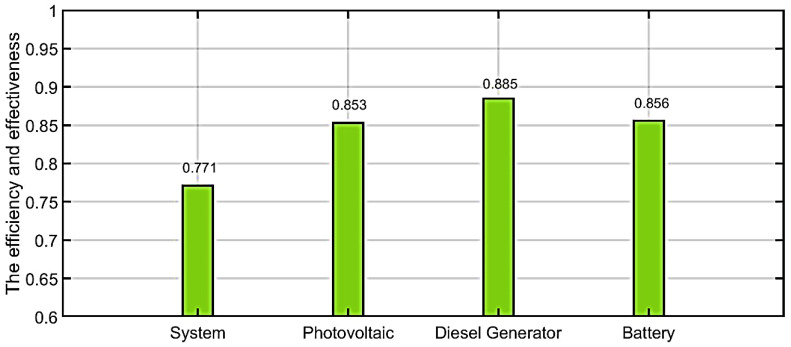
The efficiency and effectiveness by using proposed method.

**Figure 16:**
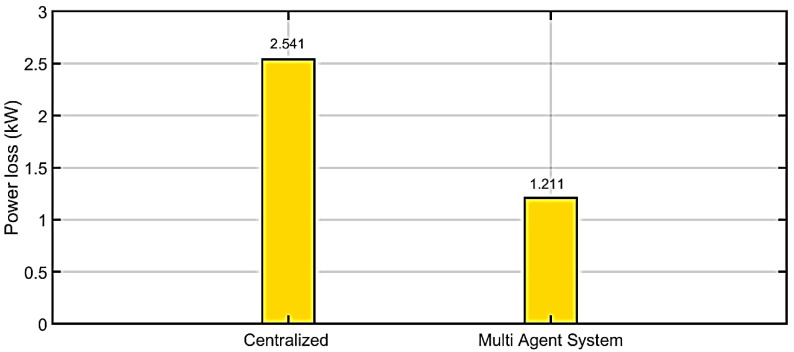
Comparison between power loss without proposed method and with proposed method.

**Figure 17:**
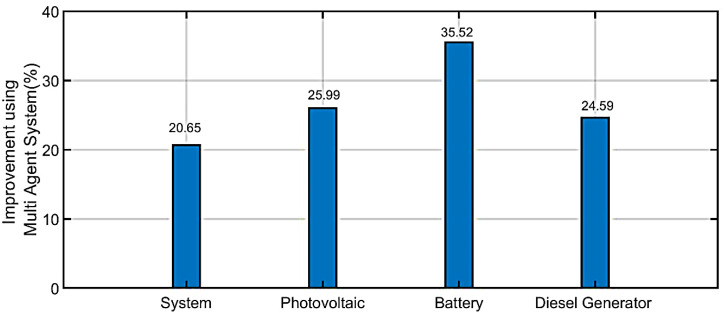
Improvement (%) by using proposed method.

The comparison between with and without corrective method is illustrated in Table 2. Figure 14 shows the efficiency and effectiveness without the proposed MAS. Figure 15 shows the efficiency and effectiveness by using the proposed method. Figure 16 shows the comparison between power loss without the proposed method and with the proposed method. Figure 17 shows the improvement (%) by using the proposed method. Table 3 shows the difference between with and without the corrective method.

## 8. Conclusions

This paper is intended to serve as a preliminary basis for quantifying the environmental and social benefits resulting from microgrid implementation. This paper presents a new cooperative controller for coordinating the multi renewable energy resources operation using the IEEE 9 test feeder as the basis and with major modifications. The suggested control scheme defines the data exchange within, and among, a multi agent system to enable MG’s flexible control in Internet of Energy. The proposed control objectives are achieved with the evaluation of the stability considering network latency. The proposed controller depends on the information transferring between the connected agents in the MG system. In addition, the reactive/active power is optimally shared among the RERs. The proposed controller improves the performance of the primary droop control method that can’t adjust the MG-*VF* to their nominal values, and also, it does not enhance the power-sharing among the RERs in MG. A hypothetical multi-agent MG system is designed to prove the proposed controller’s effectiveness using the MATLAB/Simulink environment in the presence of the different scenarios in MG. In addition, this study presents a hierarchical communication platform with a two-level structure, which is suitable for the microgrid management system. The proposed platform uses TCP/IP for local microgrid data exchange and as a backup communication method among microgrids in case of a failure in the cloud level communication. Finally, for accessing the data related to the power consumption of the individual loads, the researchers developed a reliable web portal associated with the IoT environment. The authors also provided a GUI after plotting a graph of power consumption for determining the power usage of every appliance. The results presented the effectiveness of proposed methods in equally sharing the active/reactive power of loads, during constant power load, and load change events. The controller proposed in this paper achieved savings of 20.65% for the system, 25.99% for photovoltaic, 35.52 for diesel generator, 24.59 for batteries, and 52.34% for power loss.

Future extension of this work may include the integration of the LoRaWAN network, with the proposed IoT architecture, because the use of the LoRaWAN technology could lead to a very promising solution, due to its good coverage capabilities (both in outdoor and in hybrid environments), whereas its most critical aspect is represented by the relatively low data throughput and duty cycle limitation.

## Figures and Tables

**Table 1:** Parameters of Photovoltaic.

Parameters	Values
Short-circuit current $\left(I_{\mathrm{sc}}\right)$	5.96 (A)
Parallel string	1
Maximum current $I_{\mathrm{mp}}$	5.58 (A)
Maximum voltage $\left(V_{\mathrm{mp}}\right)$	54.7 (V)
Temperature coefficient of $\left(V_{\mathrm{oc}}\right)$	−0.27269 (%/°C)
Temperature coefficient of $\left(I_{\mathrm{sc}}\right)$	0.061745 (%/°C)
Shunt resistance $\left(R_{\mathrm{sh}}\right)$	269.5934 Ω
Series resistance $\left(R_{s}\right)$	0.37152 Ω
Diode saturation curent $I_{o}$	$6.3\times 10^{-1}\;$(A)
Series connected modules	7
Short-circuit current $\left(V_{\mathrm{oc}}\right)$	6.42 (V)
Number of cells	96

**Table 2:** BUS generation and load parameters of IEEE 9 test system.

Bus	$P_{R_{i}}\;\left(p.u.\right)$	$P_{L_{i}}\;\left(p.u.\right)$	$V_{i}\;\left(p.u.\right)$	$D_{R_{i}}\;\left(s\right)$	$D_{L_{i}}\;\left(s\right)$
1	0.67	0	1	5	0
2	1.63	0	1	5	0
3	0.85	0	1	5	0
4	0	0	1	0	$10^{-2}$
5	0	0.9	1	0	2
6	0	0	1	0	$10^{-2}$
7	0	1	1	0	2
8	0	0	1	0	$10^{-2}$
9	0	1.25	1	0	2

**Table 3:** Difference between with and without corrective method.

Efficiency and Effectiveness	Proposed Method	Centralized	Percentage Improvement with Proposed System (%)
$\eta_{sys}$	0.771	0.639	20.65%
$\eta_{PV}$	0.853	0.677	25.99
$\eta_{DG}$	0.885	0.653	35.52
$\eta_{BATT}$	0.856	0.687	24.59
$P_{loss}$	1.211	2.541	52.34%

## Data Availability

Data sharing not applicable.

## Nomenclature

Abbreviations & Acronyms

Renewable Energy Resources	RERs
Networked Renewable Energy Resources	NRERs
Peer-To-Peer	P2P
Internet of Things	IoT
Internet Protocol	IP
Transmission Control Protocol	TCP
Message Queuing Telemetry Transport	MQTT
Renewable Energy Technologies	RET
Distributed Energy Resources	DER
Multi Agent Systems	MAS
Internet Of Energy	IoE
Alternating Current	AC
Microgrid	MG
Direct Current	DC
Low Voltage	LV
Business Intelligence	BI
Local Area Network	LAN
Measurement Units	MU
Identity Document	ID
Application Programming Interface	API
Photovoltaic	PV
